# New Bills of Mortality for London

**Published:** 1840-04

**Authors:** 


### NEW BILLS OF MORTALITY FOR LONDON.

IT is most gratifying to us to be able to state that the metropolis of England can, at last, boast of weekly bills of mortality of the most authentic kind, and which leave the medical statistician little to desire. For these we are indebted to the enlightened views entertained by Mr. Lister, the registrar-general of births, deaths, and marriages, who has authorized his able coadjutor, Mr. Farr, to draw the map according to the form and principles adopted by this gentleman in his admirable Report noticed in Art. II. of our present Number. A bill is printed weekly containing the deaths from all causes registered during the week ending on the Saturday immediately preceding, and is circulated very widely. The ages are classed only under three heads, viz., from birth to 15, from 15 to 60, from 60 upwards—a limitation for which, doubtless, there are good reasons, but which we cannot help greatly regretting. We think the divisions might have been, at least, doubled, if not extended to decennial periods—the first ten years being still further subdivided. We are, however, most thankful for what we have got.

The weekly bill is framed from the deaths registered in the thirty-two parochial districts into which London and its vicinity are divided. These include the City of London within and without the walls, the City and Liberties of Westminster, the Out Parishes within the bills of mortality; and the parishes of St. Mary-le-bone; St. Pancras; Kensington; Fulham; Hammersmith; St. Luke, Chelsea; Paddington; St. Mary, Stoke Newington; St. Leonard, Bromley; St. Mary-le-bow; Camberwell; Greenwich; St. Nicholas and St. Paul, Deptford; and Woolwich. For the sake of convenient comparison, the districts are classified as follows: *West districts*—Kensington; St. George, Hanover Square; Westminster; St. Martin in the Fields; St. James. *North districts*—St. Mary-le-bone; St. Pancras; Islington; Hackney. *Central districts*—St. Giles and St. George; Strand; Holborn; Clerkenwell; St. Luke; East London; West London; City of London. *East districts*—Shoreditch; Bethnal Green; Whitechapel; St. George in the East; Stepney; Poplar. *South districts*—St. Saviour; St. Olave; Bermondsey; St. George, Southwark; Newington; Lambeth; Camberwell; Rotherhithe; Greenwich. The population of the whole, according to the enumeration of 1831, was 1,594,890.

It is our intention henceforward to give, in detail, quarterly abstracts of these tables, arranged, probably, according to the seasons. These will soon constitute a series of authentic statistical documents, such as have never yet appeared in this country, and fit to form the basis of the most momentous hygienic conclusions. These abstracts we shall commence in our next Number, contenting ourselves, at present, with giving the gross results of the first two months of the present year, which are as follows:

| | | *January.* Week ending 11th, | 18th, | 25th | *February.* Week ending 1st, | 8th, | 15th, | 22d, | 29th | Total. |
|---|---|---|---|---|---|---|---|---|---|---|
| Weekly Deaths in the | West Districts ... | 149 | 160 | 122 | 108 | 125 | 98 | 121 | 138 | 1021 |
| | North Districts... | 171 | 170 | 163 | 157 | 152 | 145 | 141 | 192 | 1291 |
| | Central Districts.. | 199 | 230 | 182 | 187 | 157 | 172 | 184 | 170 | 1481 |
| | East Districts...... | 225 | 216 | 207 | 200 | 167 | 190 | 199 | 171 | 1575 |
| | South Districts ... | 223 | 221 | 242 | 183 | 217 | 208 | 210 | 245 | 1749 |
| | Total............ | 967 | 997 | 916 | 835 | 818 | 813 | 855 | 916 | 7117 |
| Weekly Deaths at different ages, viz., from ........ | 0 to 15............ | 404 | 392 | 384 | 360 | 346 | 356 | 345 | 375 | 2962 |
| | 15 to 60........... | 332 | 361 | 302 | 291 | 278 | 282 | 319 | 330 | 2495 |
| | 60 and upwards... | 230 | 244 | 230 | 182 | 194 | 174 | 189 | 211 | 1654 |
| | Total............ | 966 | 997 | 916 | 833 | 818 | 812 | 853 | 916 | 7117 |

